# Decontamination protocols affect the internal microbiota of ticks

**DOI:** 10.1186/s13071-023-05812-2

**Published:** 2023-06-07

**Authors:** Natalia Fernández-Ruiz, Sophia Pinecki-Socias, Agustín Estrada-Peña, Alejandra Wu-Chuang, Apolline Maitre, Dasiel Obregón, Alejandro Cabezas-Cruz, Ignacio de Blas, Ard M. Nijhof

**Affiliations:** 1 Faculty of Veterinary Medicine, University of Zaragoza, 50013 Zaragoza, Spain; 2 Group of Research on Emerging Zoonoses, Instituto Agroalimentario de Aragón (IA2), 50013 Zaragoza, Spain; 3 Institute of Parasitology and Tropical Veterinary Medicine, Freie Universität Berlin, 14163 Berlin, Germany; 4 Anses, INRAE, Ecole Nationale Vétérinaire d’Alfort, UMR BIPAR, Laboratoire de Santé Animale, 94700 Maisons-Alfort, France; 5 School of Environmental Sciences, University of Guelph, Guelph, ON Canada; 6 Veterinary Centre for Resistance Research, Freie Universität Berlin, 14163 Berlin, Germany

**Keywords:** Ticks, Microbiota, Bleach, External contamination

## Abstract

Studies on the microbiota of ticks have promoted hypotheses about the combined effects of the bacterial community, its functional contributions to the tick’s physiology or probable competition effects with some tick-borne pathogens. However, knowledge on the origin of the microbiota of newly hatched larvae is missing. This study aimed to elucidate the source(s) of the microbiota in unfed tick larvae, addressing the composition of the “core microbiota” and the best ways to decontaminate eggs for microbiota studies. We applied laboratory degree bleach washes and/or ultraviolet light treatments on engorged *Rhipicephalus australis* females and/or their eggs. No significant effects of these treatments on the reproductive parameters of females and the hatching rates of eggs were observed. However, the different treatments did show striking effects on the composition of the microbiota. The results indicated that bleach washes disrupted the internal tick microbiota in females, implying that bleach may have entered the tick and subsequently affected the microbiota. Furthermore, the analyses of results demonstrated that the ovary is a main source of tick microbiota, while the contribution of Gené’s organ (a part of the female reproductive system that secretes a protective wax coat onto tick eggs) or the male’s spermatophore requires further investigation. Further studies are needed to identify best practice protocols for the decontamination of ticks for microbiota studies.

**Graphical Abstract:**

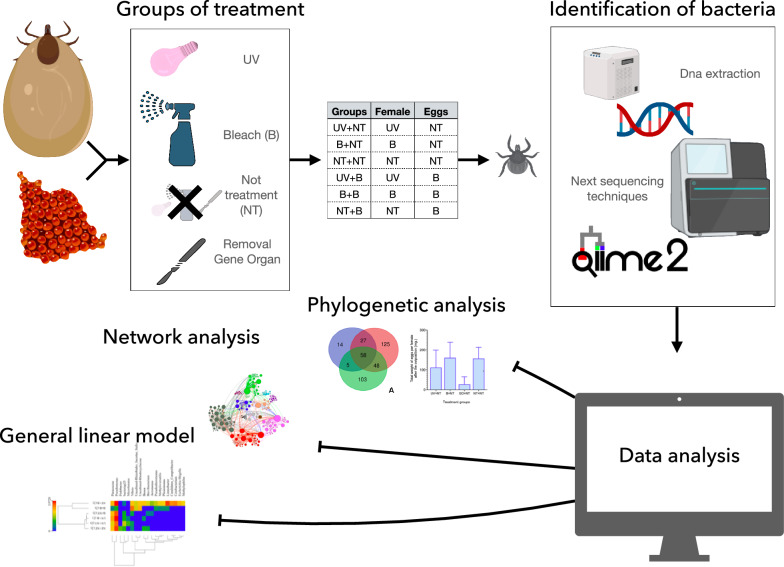

**Supplementary Information:**

The online version contains supplementary material available at 10.1186/s13071-023-05812-2.

## Background

Microbiota is defined as the specific set of bacteria, viruses, fungi or protozoans found in a specific environment [1]. In recent years, many studies pinpointed that microbiota plays an essential role in the physiology of a range of arthropods [2, 3]. Ticks are obligate hematophagous ectoparasites, which is believed to influence the composition of its microbiota [4] because of changes introduced by the blood meal and the possible influences from the environment while questing for or feeding on a host. It has been demonstrated that both abiotic (temperature, landscape) and biotic factors (host blood composition) may shape the composition of the tick’s microbiota [5–7]. Despite significant differences in the microbiota composition observed between different tick species and studies, some patterns in the bacterial composition have emanated [8–11].

Several studies demonstrated the effects produced by specific bacteria on ticks [12, 13]. It has for instance been revealed that bacteria could affect the tick’s physiology by affecting tick reproduction, feeding, nutrition or adaptation to abiotic conditions, among others [14]. As the tick’s diet is restricted to blood, this also results in the lack of some vitamins and cofactors [15], and several detected symbionts have been associated with providing these nutrients to ticks [16]. For example, the association of ticks with a *Coxiella*-like symbiont seems to have evolved to provide the tick with B vitamins and cofactors [17]. Similarly, *Francisella*-like symbionts have been reported to be involved in metabolic pathways regarding the synthesis of B vitamins such as biotin, folic acid and riboflavin [18, 19]. In addition, there is empirical evidence that some tick-borne pathogens may interact in the tick with some other bacterial taxa [10, 20, 21]. It is for instance known that the presence of *Anaplasma phagocytophilum* induces the expression of an anti-freezing protein in *Ixodes scapularis*, increasing the survival rate of ticks at cold temperatures [22]. However, studies on tick microbiota are still fragmented compared to those of other arthropods (e.g. *Drosophila melanogaster*) for which details on symbionts, molecular functions and assemblages of bacterial communities are available [23, 24]. We are only just beginning to understand the intricate relationships between bacterial communities and ticks.

Several studies addressed protocols to remove the external contaminants of ticks (e.g. from the vegetation), which is required for “clean” sequencing [25, 26] to analyse the microbiota interacting with the tick. It has been reported that ethanol-based methods are inefficient to eliminate the bacterial DNA on the tick’s surface, producing “noise” in the sequencing results [27]. Several studies recommended the use of sodium hypochlorite as the best method to remove external contaminations. However, alternative external sterilisation methods have not been extensively tested, and the source of a hypothetical core microbiota remains only suspected [9, 28, 29]. This core microbiota could be analysed only after a thorough removal of contaminating bacteria.

This study aimed to identify the main sources for the tick larval microbiota and to identify the primordial set of bacterial taxa present in newly oviposited eggs. Two methods were used to remove potential contamination sources: (i) a 1% dilution of laboratory degree bleach and/or (ii) ultraviolet light, applied to either engorged females or eggs from the same (un)treated female(s). The DNA was extracted from larvae resulting from combinations of treatments, and the bacterial composition of the microbiota was identified by 16S ribosomal RNA (rRNA) sequencing. DNA was also extracted from the ovary and the removed Gené’s organ of engorged females (treated and control) to compare the microbiota composition between these organs. A secondary focus was the evaluation of the soundness of the decontamination methods in removing potentially contaminating bacteria.

## Methods

### Background

Since we assumed that contamination of the eggs with DNA from the female’s cuticle could affect the microbiota detected in the larvae, our first aim was to remove these hypothetically contaminating bacteria. We can only make inferences about the probable source(s) of the microbiota after observing and comparing organs and after treatments of engorged females and/or eggs. We evaluated the use of bleach and ultraviolet light as decontaminating agents. As an additional source of comparison, we ablated the Gené’s organs of a few females; this organ is a part of the female reproductive system that coats the eggs with a protective wax layer, protecting the eggs from desiccation. The removal of Gené’s organ causes the desiccation and death of the eggs [30], but we nonetheless removed it in females of one group to examine if the organ could provide bacterial taxa to the eggs.

### Experimental design

Ticks used in this study were engorged *Rhipicephalus australis* females, original strain Parkhurst from a laboratory colony. Ticks were individually weighed and incubated in sterile individual flasks. After the females were removed from the flask at the end of oviposition, each flask was weighed to further extrapolate the weight of each egg mass with minimal disturbance to eggs inside the flask. A total of 56 engorged females that had fed on the same batch of hosts were used to avoid variation introduced by different blood meal origins. These female ticks were allocated into four different groups (*n* = 14), kept at 27 °C and 80% relative humidity. The treatments were (i) ultraviolet light (“group UV”), (ii) sodium hypochlorite at 1% (laboratory quality bleach, “group B”), (iii) untreated (“group NT”), and (iv) females with Gené’s organ surgically removed (“group GO”).

**Fig. 1:**
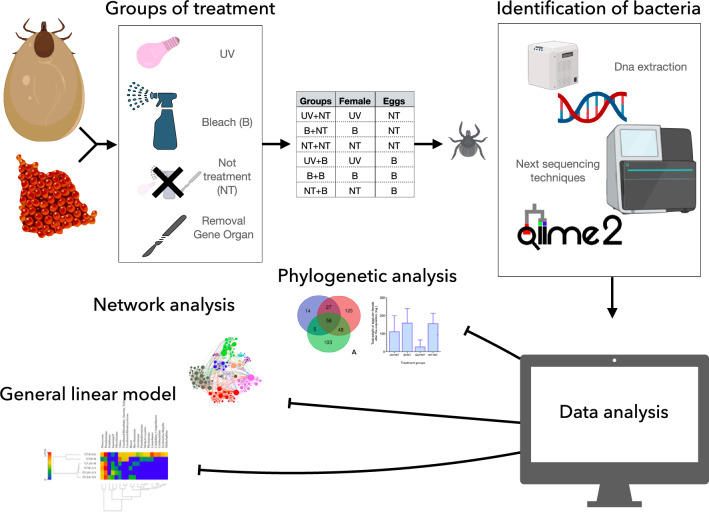
Schematic view of the workflow. The figure shows the three main groups of treatments on ticks, the delivery of further groups of eggs with/without treatment and the obtention of the final data through next generation sequencing

Once the oviposition was finished, the eggs from each female were allocated into two batches. One was further treated with sodium hypochlorite at 1%; the other was left untreated as a control, thus obtaining a total of six groups of eggs (see point 3, below). Eggs coming from the GO group were also kept under the same conditions but died and were not considered further. The different groups of eggs were weighed and incubated under identical conditions until hatching. Larvae from each group of eggs were prepared for DNA extraction. A schematic view of the experimental design is shown in Table 1 and Fig. 1.

**Table 1:** Schematic table with the different groups in the experiment

Group	Females	Eggs
UV + NT	Ultraviolet light	No treatment
B + NT	Bleach	No treatment
NT + NT	No treatment	No treatment
UV + B	Ultraviolet light	Bleach
B + B	Bleach	Bleach
NT + B	No treatment	Bleach

Six groups are classified with the treatment that receive the females and eggs. In this study, there are three possible treatments UV (ultraviolet light), B (bleach) and NT (no treatment)

### Laboratory treatments of engorged females and eggs

For the UV group, females were exposed to ultraviolet light in a flow chamber, 1 h on the ventral surface followed by 1 h on the dorsal surface. This treatment required minimal manipulation of each tick, and UV exposure is a common laboratory sterilisation procedure. The females of group B were treated with sodium hypochlorite at 1% (diluted from laboratory-quality sodium hypochlorite and sterile milliQ water). Females were washed three consecutive times and then subjected to three consecutive cleaning washes with sterilised water for 30 s each [27]. The groups NT and GO did not receive any treatment and were immediately placed in the incubator for oviposition. As soon as the females started to lay eggs in the GO group, Gené's organ was surgically removed under a stereomicroscope and discarded; each treated female was introduced into a new sterilised flask after the removal of the organ.

After the oviposition of the females of every group except GO, the eggs of each female were allocated into two groups. Approximately 50% of the eggs of each female from B, UV or NT groups were treated with sodium hypochlorite as stated before for the females. The remaining half of the eggs was left untreated. We thus obtained six groups of eggs, namely “UV + NT”, “UV + B”, “B + NT”,” B + B”, “NT + B”,and “NT + NT” (the denomination includes the treatment of females followed by treatment of eggs) that were subsequently left to incubate further under the same conditions. Once all larvae had hatched, the hatching ratio was estimated, and DNA was extracted from the different groups of larvae. We also removed the ovary and Gené's organ of 1/3 of the treated and control females.

We recorded the weight of the engorged females, eggs and larvae to test the influence of the treatments on the reproductive performance of each treatment (measured as the average weight of eggs and the larval hatching ratio). The hatching ratio of the eggs was performed semi-quantitatively, using four percentage divisions of 100–75%, 75–50, 50–25 and 25–0% hatching. The reproductive parameters were analysed using Fisher's analysis of variance (ANOVA).

### DNA extraction

Genomic DNA was extracted from pools of around 30 larvae per replicate using the Nucleospin DNA Insect kit (Macherey-Nagel, Düren, Germany) following the manufacturer’s protocol, resulting in a total of 48 larval samples and 12 samples of ovaries and Gené’s organ that were extracted using the Nucleospin XS Tissue kit from Macherey-Nagel. Three controls for each DNA extraction batch were introduced. Control samples contained all chemicals used in the DNA extraction. After the extraction, the DNA quality from eight samples of each group was measured using a NanoDrop™ One (Thermo Scientific, Waltham, MA, USA). Samples with > 900 ng of DNA at ≥ 20 ng/μl were sent to Novogene Bioinformatics Technology Co. (London, UK) for amplicon sequencing of the bacterial 16S rRNA gene using universal primers 515F/806R targeting the V4 variable region of the 16S rRNA as recommended by the Earth Microbiome Project (https://earthmicrobiome.org/).

### 16S rRNA sequence processing

The analysis of 16S rRNA sequences was performed using QIIME 2 pipeline v. 2019.1 [31]. The sequences were denoised and merged using DADA2 software [32] as implemented in QIIME 2. The amplicon sequence variants (ASVs) were aligned with the q2-alignment of MAFFT [33] and used to construct a phylogeny with q2-phylogeny of FastTree 2 [34]. Taxonomy was assigned to ASVs using a classify-learn naïve Bayes taxonomic classifier [35], based on the SILVA database (release 138) [36]. After the query in QIIME 2, we used the package “decontam” [37] from Bioconductor [38] to remove the contaminants from the DNA extraction [39].

### Data analyses

#### Characterisation of the core microbiome

Bacterial taxonomic profiles were collapsed at genus level and above for analyses. Rare taxa were removed for the data set (< 10 count; < 10% prevalence). Venn diagrams were used to quantify the number of taxa shared among larvae coming from different treatments and among larvae, ovary and Gené’s organ. The analyses were performed using the web tool available from Ghent University (Belgium) https://bioinformatics.psb.ugent.be/webtools/Venn/ (accessed August 2022). The core microbiome analysis was calculated involving all samples and was identified at different prevalence from 20 to 100%. The analysis was performed using the web tool MicrobiomeAnalyst https://www.microbiomeanalyst.ca [40].

#### Diversity and differential taxonomic composition analyses

We used Faith’s phylogenetic distance (PD) [41] on the log-normalised number of reads of bacterial taxa to calculate the phylogenetic microbial richness from each treatment; significant differences between groups were calculated using a Kruskal-Wallis test. Beta diversity analysis was conducted based on Bray–Curtis dissimilarity distance [42].

Once we examined the alpha and beta diversity of the microbiota in larval ticks from different treatments, we aimed to determine which taxa were responsible for the observed differences. Statistical analyses were carried out with IBM SPSS Statistics 19.0 for Windows (IBM Corp., 2019). A generalised linear model (GLM) was used to assess the effect of the different treatments on the larval microbiota, discerning between the treatments on females and eggs. The GLM was built over the normalised reads of taxa using Aitchison’s log-ratio transformation, which is applicable to compositional data [43]. Since a log transformation cannot be applied to zeros, we adhered to the usual approach (known as “pseudocounts”) of replacing the “0’s” by “1’s”, thus giving the result of “0” after log transformation. Initially, the statistical analysis of the number of reads was carried out using a Kolmogorov-Smirnov test to determine the normal distribution of data. A Kruskal-Wallis test was used to determine the effects of the specific bacteria of the microbiota. We used a complete factorial design with interactions between factors (UV, B and NT). Duncan’s post hoc test was used to evaluate the effects on treatment groups. The level of significance was set at *P* < 0.05 [44].

#### Microbial network analyses

We used graph constructs to study the effect of the different treatments on the topology of the microbial interactions among treated and control pools. A network is a set of nodes interconnected by edges. The nodes represent the bacterial genera; the edges are the correlation between the number of reads of each taxa for each treatment. The number of reads of each taxon was used in a sparCC correlation between every pair of nodes as a measure of the “strength of the link” between every two specific taxa. We used a previously published method [45] implemented in R in the package SpiecEasi [46]. The software Gephi 0.92 was employed for other network calculations (http://www.gephi.org, last accessed February 2022). We used several indices characterising each network, namely the weighted degree, betweenness centrality and modularity, as well as the Louvaine algorithm (as included in Gephi 0.92) to calculate the modularity; this is a measure of the clustering of interacting nodes, detecting groups of nodes that interact statistically more frequently among them than with the rest of the nodes.

We tested the resilience of the networks resulting from each treatment to systematic node removal, by either a random attack with 100 iterations or a directed attack, removing the nodes according to their value of betweenness centrality (the highest, the first). A third method involved the “extinction in cascade”, which measures the effect on the network of the removal of hypothetical keystone bacteria. This is important as it is expected that many nodes will be correlated with the keystone taxa and therefore appear correlated with the latter. The removal of keystone taxa will be left as a signature in the network in which the deletion of a few taxa will remove many secondary taxa correlated with them. The analysis of the network resilience was done with the package NetSwan for R [47].

## Results

### Treatments of females did not affect their reproductive efficiency

**Fig. 2:**
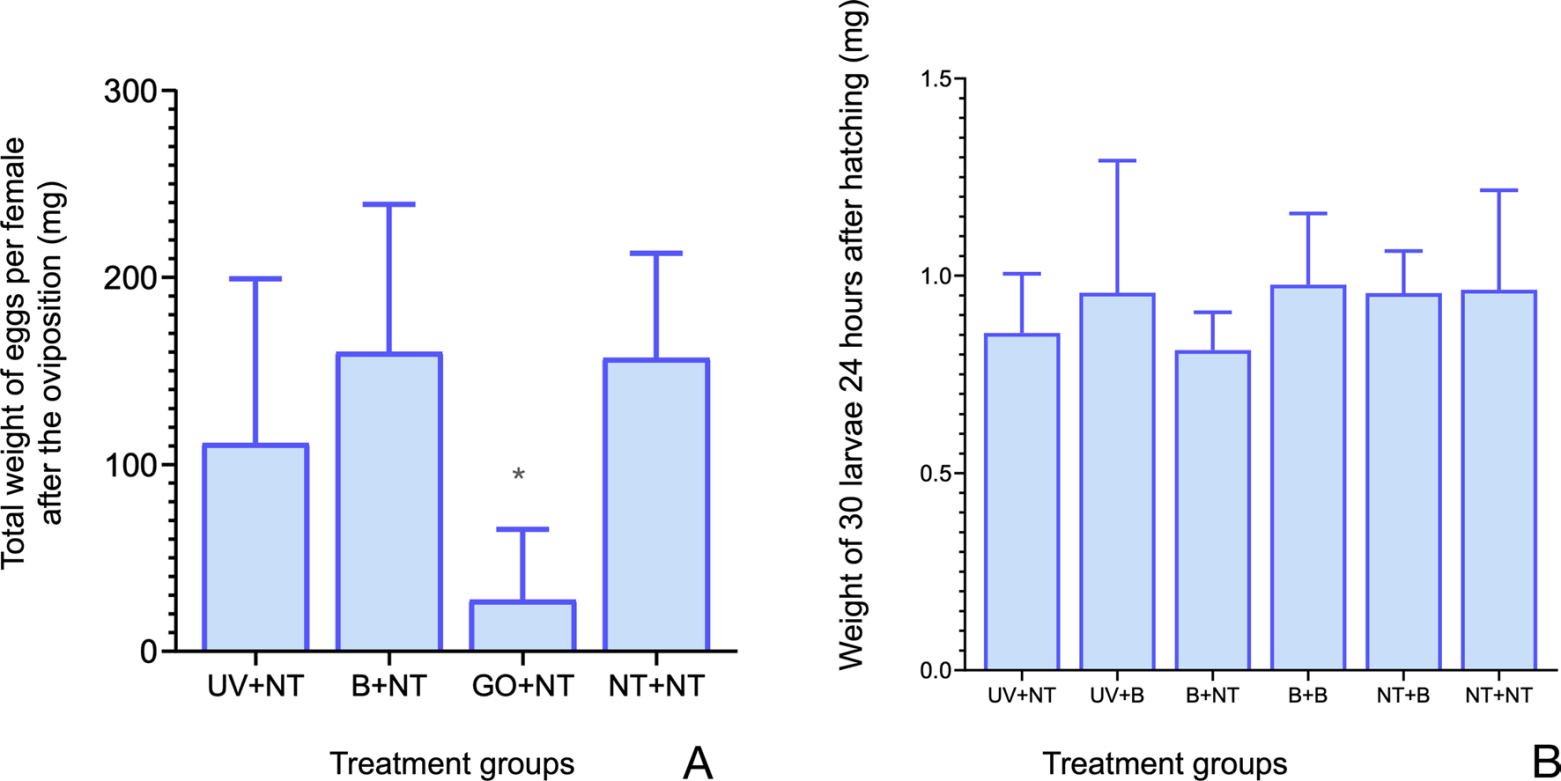
Values and statistical differences of the reproduction success of treated females and/or eggs versus untreated ones. **A** Mean and standard deviation of egg’s weight after the oviposition from the different female treatment groups. **B** Mean and standard deviation of the weight of 30 larvae after hatching from the different female and/or eggs treatment groups. *P-value ≤ 0.05

The treatment of the females with either bleach or UV light did not affect the reproductive fitness of the females, as evidenced by the similar weights of egg batches laid by females of different treatment groups (Fig. 2A). The only treatment impacting the weight of the eggs was the removal of Gené’s organ. The hatching rate was close to 100% in all groups, and there were no significant differences in the weight of the hatched larvae (Fig. 2B). Results of this part of the study showed that the reproductive success of the UV- or bleach-treated females or eggs was not altered compared to the control group.

### Treatments interfere with microbiota in diverse ways

**Fig. 3:**
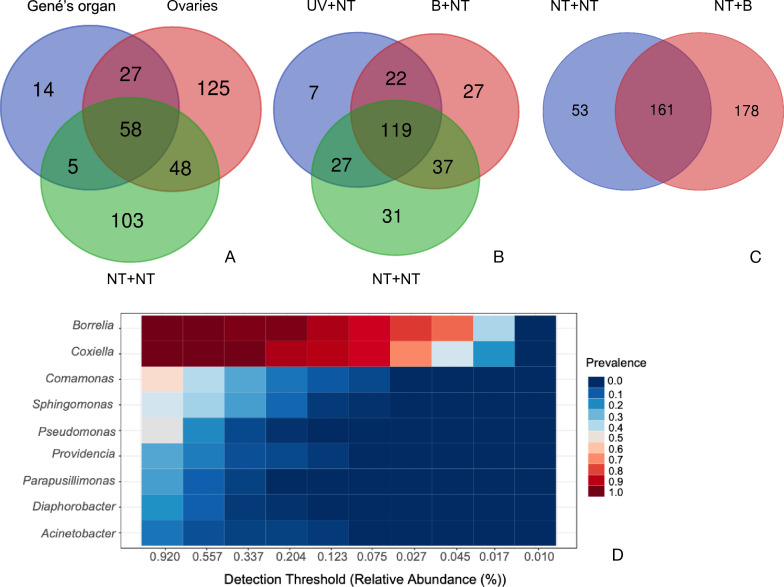
Comparative overview of the sharing of bacterial taxa among groups of treatments in females and/or tick eggs. **A** Venn diagram showing the number of bacteria shared among the larvae, ovary and Gené’s organ in the group of females without treatment for removal of external microbiota. **B** Venn diagram showing the number of bacteria shared between the larvae of groups of treated engorged female ticks (the “NT” denomination for eggs is included in the labelling). **C** Venn diagram showing the number of bacteria shared between the groups of larvae whose eggs received different treatments. **D** The core microbiota of the studied larvae. Core bacterial taxa are in Y axis, with an expected detection threshold (percent of relative abundance) in the X axis; the legend shows the prevalence of each taxon in the total of the studied samples

Sequencing protocols revealed a total of 424 bacteria in the whole set of treatments and samples, including the ovary, Gené’s organ and larvae. This included the detection of a *Borrelia* sp., probably *Borrelia theileri*, in all groups. The presence of this bacterium could change the total number of reads in the samples making some other taxa under-represented. However, all the analysed samples were positive to *Borrelia* sp.; therefore, results remain comparable. The number of bacterial taxa shared by treatments and sample is shown in Fig. 3. The relative contribution of both ovary and Gené’s organ to the NT + NT larvae is of interest. The ovary alone made a contribution of 48 taxa (12% of total) to the larval microbiota (Fig. 3A). To note, 125 (32%) and 103 (27%) taxa were detected only in ovary or larvae from untreated females, respectively. This demonstrates that the microbiota present in the ovary is not transferred *in toto* to the larvae and that the 103 taxa exclusively found in the larvae may have originated from another source. Of the taxa, 27.7% were shared between Gené’s organ and the ovary of NT females. Larvae originating from treated females from which the eggs were left untreated (B + NT, UV + NT and NT + NT) shared up to 119 of the 270 taxa (44%) identified (Fig. 3B). The treatment of eggs with bleach increased the number of bacterial taxa and the number of reads in larvae (Additional file 1: Table S1). According to these data, the eggs treated with bleach (NT + B group) carried more bacteria than those left untreated but also originating from untreated females (NT + NT group, Fig. 3C). This could be explained by a change of the relative abundance of the bacterial taxa in treated groups: if prominent bacteria are eliminated from the sample by the bleach, then the remaining taxa appear to be more abundant by the artifact derived from the fixed number of total reads assumed in the sequencing methodology. The core microbiome is shown in Fig. 3D, together with the prevalence and the taxonomic profile. For example, *Borrelia* and *Coxiella* are presented in all samples with a high prevalence, while *Comamonas* and *Pseudomonas* only appeared in between 50 and 60% of samples.

**Fig. 4:**
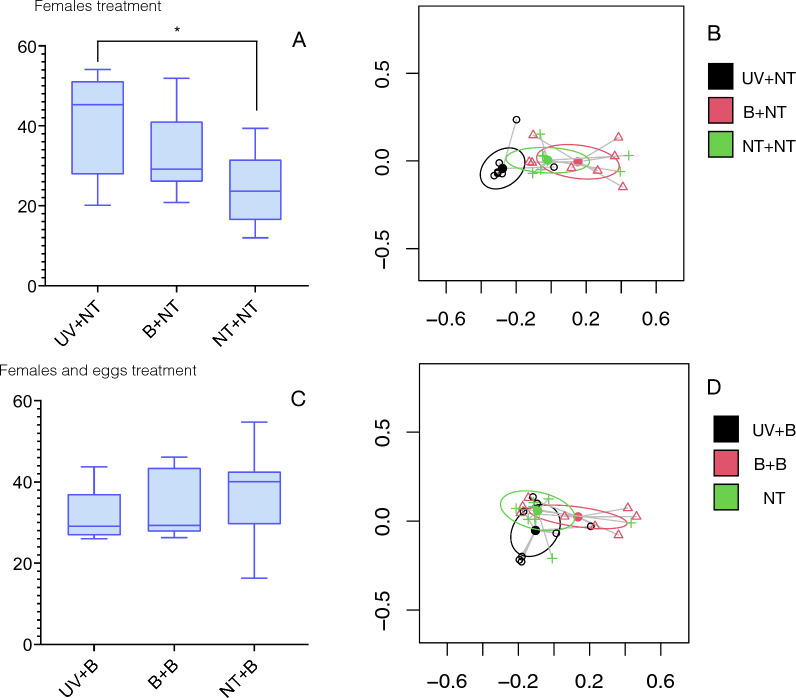
Measures of phylogenetic diversity of the studied set of samples. **A** Faith index of phylogenetic diversity of the microbiota of larvae from females receiving a treatment for removal of external microbiota. Significant results appear only between UV + NT and NT + NT with a *P*-value of 0.02. **B** Bray-Curtis index of the microbiota of the larvae coming from treated females. Significant results appear only between UV + NT/NT + NT and UV + NT/B + NT (*P* = 0.005 and *P* = 0.002). **C** Faith index of phylogenetic diversity of the microbiota of the larvae in which engorged females and eggs received a treatment. **D** Bray-Curtis index of the microbiota of the larvae coming from females and eggs treatment

The alpha and beta diversity of the microbiota of the larvae is displayed in Fig. 4. Significant differences in PD resulted from comparisons between UV + NT and NT + NT groups (*P* = 0.02, Fig. 4A). Other groups showed no significant differences of alpha diversity. However, the microbiota of larvae from females of the UV group was phylogenetically different to the NT pool. The differences in Bray-Curtis distance were significant between UV + NT/NT + NT and UV + NT/B + NT groups (*P* = 0.005 and *P* = 0.002, respectively, see Fig. 4B). This is an important finding because it means that the number of reads of bacteria in the larvae was significantly different after the UV treatment. The results involving the pools whose females and eggs were treated (UV + B, B + B, NT + B) are included in Fig. 4C and D, displaying a lack of significant differences for both Faith’s PD and Bray-Curtis between pairs of groups. Therefore, the only treatment significantly affecting the tick’s microbiota was the UV exposure to females (UV + NT). Treatments involving bleach on females and/or eggs did not result in changes in the phylogenetic diversity.

### Bacterial taxa affected by treatments are internal to ticks

**Fig. 5:**
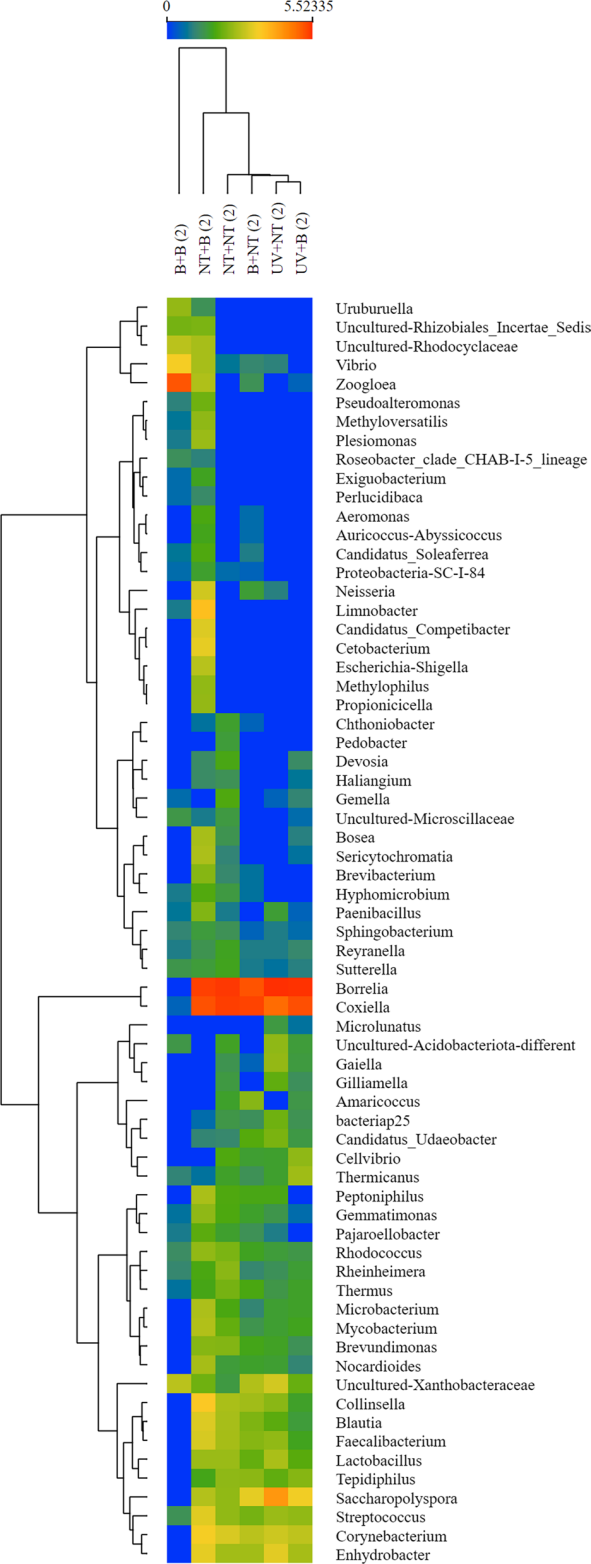
A heatmap of the number of reads of the bacteria that resulted significantly different in the treatment groups, according to a general linear model. Treatments are included at the top (columns) and taxa at right (rows). In the preparation of the heatmap, taxa have been re-sorted to produce a dendrogram (left) of similarities

The plots of the normalised number of reads are shown in Fig. 5 for the taxa with significant differences among the treatments. It could be observed that the bleach treatment of females negatively affected the number of reads for bacteria such as *Borrelia*, which is considered present only in the internal tick milieu (Fig. 5).

**Fig. 6:**
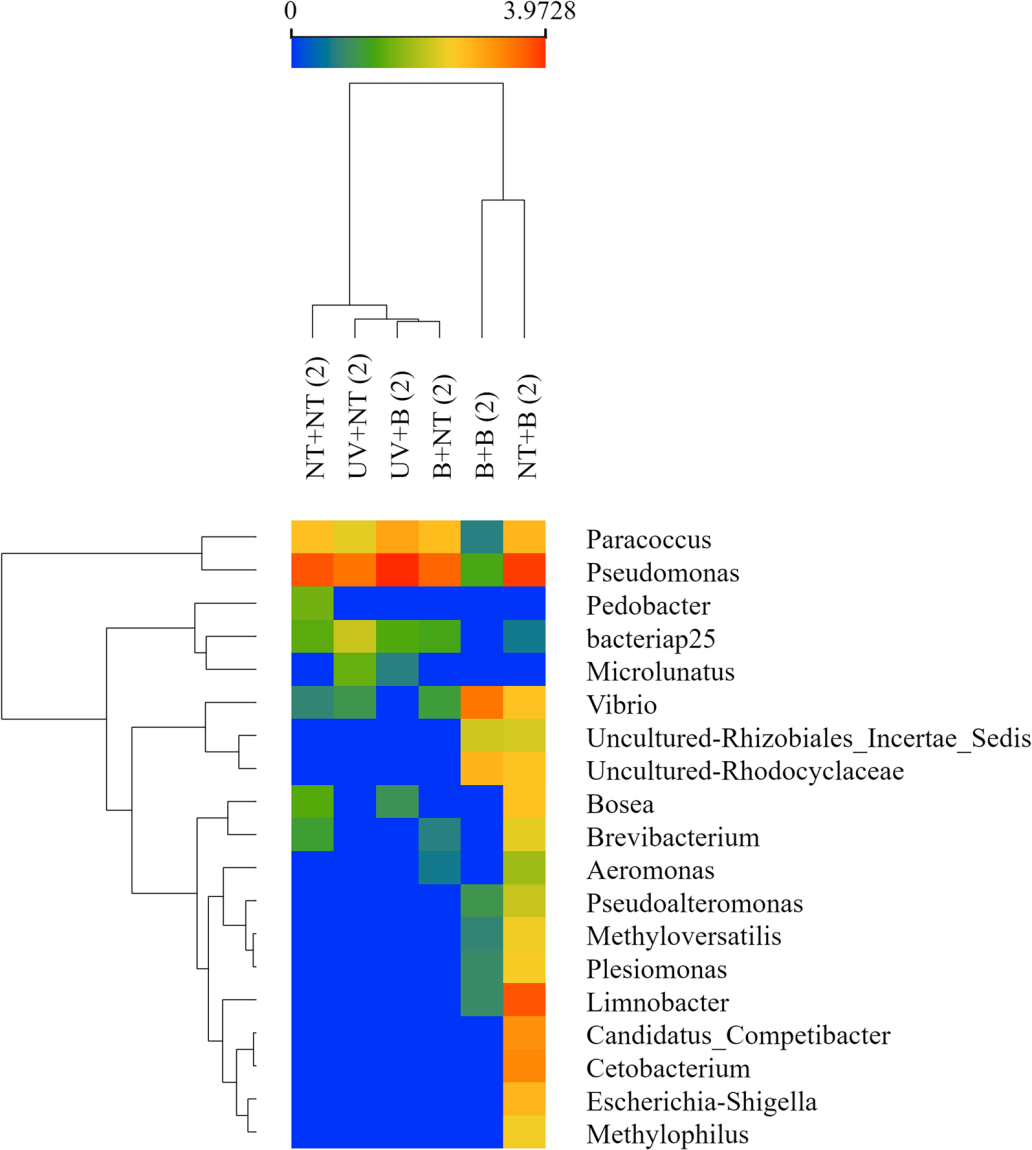
A heatmap of the number of reads of the bacteria that resulted significantly different in the treatment groups, according to a general linear model. Treatments are included at the top (columns) and taxa at right (rows). In the preparation of the heatmap, taxa have been re-sorted to produce a dendrogram (left) of similarities

Regarding the effect of treatments of eggs on the number of bacterial reads, all the taxa with significant changes are usually also present in earth and water, like *Vibrio* spp., *Pseudomonas* spp. or *Pedobacter* spp. (Fig. 6). Notably, data were normalised and the differences on extremes did not appear clearly defined in Figs. 5 and 6. Figure 5 shows that treatments on females generate clusters of significant changes in microbiota, according to the dendrogram resulting from plotting the results based on the normalised number of reads. One of the branches represents bacteria that are internally present in ticks, like *Coxiella* or *Borrelia*; the other branch includes bacteria that have an external location in tick like *Pseudomonas* spp. or *Vibrio* spp*.*

### Resilience of co-occurrence networks resulting from the treatments

**Fig. 7:**
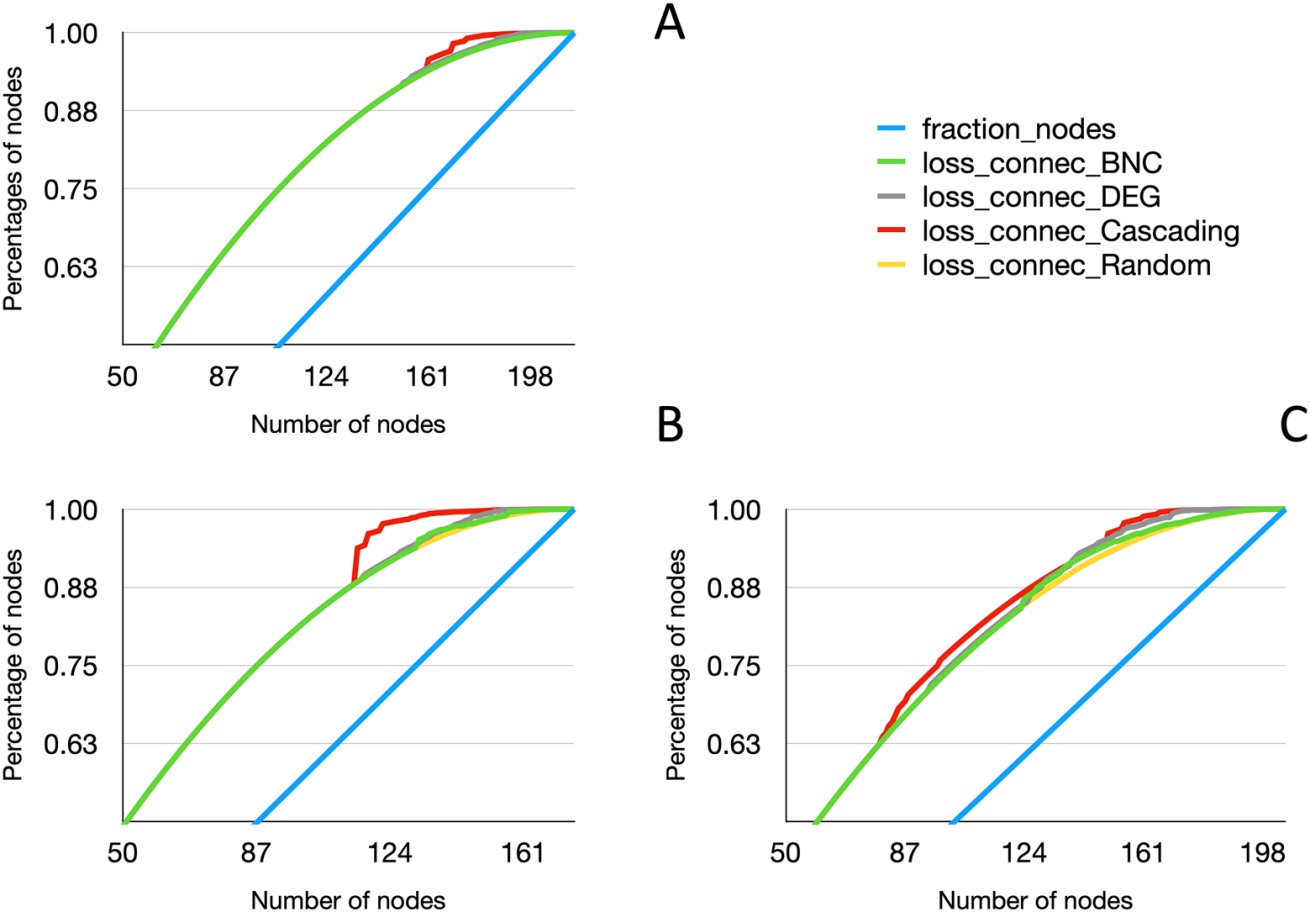
Resilience of the networks of co-occurring bacteria of the tick microbiota. The networks were produced with co-occurring bacteria in each treatment according to the number of reads (see Methods) and were subjected to the removal of nodes according to different strategies. Nodes (taxa) were removed according to centrality (BNC), weighted degree (DEG) and cascading effect (cascading) and by simple random removal of nodes. **A** The losses of resilience in the female treatment group NT + NT. **B** The losses of resilience in the female treatment group UV + NT.  **C **The losses of resilience in the female treatment group B + NT

We evaluated the connectivity of the networks of interacting bacteria using the data obtained from treatments on engorged females. We included the impact on the network’s connectivity after the random removal of taxa, the removal according to centrality indexes and the cascading effect of the secondary losses of taxa after the removal of a component of the network. Results show that lack of connectivity by cascading losses is the most obvious; it is clearly marked in larvae coming from group B + NT (female receiving a bleach treatment) (Fig. 7C). Other networks have a negligible lack of connectivity under every strategy of attack Fig. 7A and B.

## Discussion

This study was primarily intended to capture the sources driving to the primordial tick microbiota, testing different protocols for removing possible sources of external contamination. We used decontamination protocols including bleach as previously recommended [27, 48] and/or UV exposure on females and/or eggs. The reproductive success of the treated females was similar to that of treated ones, indicating that the protocols did not negatively affect tick reproduction. However, we found contrasting results regarding the best protocol to be applied on females and/or eggs to produce larvae on which to investigate a basic set of bacterial taxa.

An interesting finding was the detection of *Borrelia* reads, possibly from *B. theileri*, a poorly characterised bacterium that is widely distributed in one-host *Rhipicephalus* ticks and is perpetuated in transmission cycles between ticks and cattle through transovarial transmission in ticks. *Borrelia* reads were previously also detected in another study analysing the bacterial diversity in *Rhipicephalus microplus* ticks from a laboratory colony [49]. Its presence might have an impact on other bacteria, as previously described for other *Borrelia* spp. that were shown to affect the microbiota composition [50]. In our study, it could be used as a marker for the effect of different treatments on internal microbiota.

The most frequently used decontamination methods of external surfaces of ticks are based on ethanol or bleach [48, 51–53]. It has been reported [27] that ethanol fixes bacterial DNA on the external surface of ticks; sources can include bacteria existing on the skin or hair of hosts on which they feed or the environment in which they quest. However, published procedures regarding studies of the microbiota in arthropods and their decontamination show large variations in terms of the time of treatment, bleach concentration and its chemical quality (laboratory degree sodium hypochlorite at 1 and 10% or a commercial brand between 1 and 5%). In other words, there is not yet a “gold standard” for these decontaminating treatments, which are otherwise carried out by researchers in the field at variable rates [54, 55].

Both the index of Faith’s phylogenetic diversity and a PERMANOVA based on a Bray-Curtis distance matrix showed that large differences in larval tick microbiota exist in specimens treated with bleach compared with UV-treated or control ticks. Treatments with bleach on engorged females could generate a dysbiosis of the bacterial communities, with an equivocal effect on the internal microbiota of the resulting larvae. Other than changes in alfa and beta diversity, we observed an impact detected as the lower resilience of networks of co-occurring bacteria in bleach-treated specimens. This can be only interpreted as the persistence of a few bacterial taxa with which many of other taxa co-occur; the systematic “removal” of these “keystone taxa" in the networks drove to the elimination of the many co-occurring bacteria because of high nestedness [56, 57]. In the bacterial networks obtained for untreated ticks, however, keystone taxa are absent, and bacteria co-occur with many other taxa, the removal of which slightly affect the structure of the network [58]. The existence of these keystone taxa is easily observed in networks by the recorded increase in the number of reads of some bacteria. They could correspond well with the detection limit imposed in sequencing: the removal of prominent taxa would leave a “sample space” for rare taxa to be detected, co-occurring with other bacteria.

We hypothesise that the bleach may have entered the body of the female tick, thereby affecting internal bacteria in a variable way, even if only three relatively short immersions were carried out. This is supported by the large changes in the number of reads of “internal” bacteria, such as *Coxiella* and *Borrelia*, which were not expected to be affected by an “external” effect of the bleach. The protocols of this study were not designed to identify how the bleach may have entered the tick’s internal cavity, but the mouthparts seem to be a candidate because of the hydration processes of the tick [59]. Since the outermost portion of the cuticle is a dense layer of hydrophobic hydrocarbons as in other arthropods [60, 61], the direct entry of a solution appears to be less likely. In any case, results supported an unexpected finding on tick microbiota that should be studied before claims about the ideal decontaminating protocol are made.

While counterintuitive, the use of bleach on tick eggs originating from untreated females seems not to affect the resulting larval microbiota. Results of the GLM show that bacterial taxa considered as “internal” or even symbionts (e.g. *Coxiella*) displayed no significant changes in the number of reads, a finding supported by both Faith’s PD and PERMANOVA. We assume that the lipid layer surrounding the eggs is hydrophobic enough to prevent the entry of bleach into the eggs, therefore being a candidate for removal of contaminating bacteria on the eggs surface. Notably, results from the GLM demonstrated that only bacteria commonly associated with the environment [62, 63] or water [64, 65] were significantly altered in these experiments, suggesting that bleach did affect the microbiota of the eggs’ surface but not the set of bacteria inside them. We encourage a deeper study on the topic before accepting this protocol as a standard.

In any case, there is a fundamental question behind these results: since larval ticks were shown to share bacterial taxa with both the ovary and Gené’s organ, these bacteria are unlikely to be considered contaminants. Gené’s organ is deep inside the female tick and only protrudes when oviposition begins [64], a process in which external contamination is unlikely in our protocols because all observations were made in a sterile environment. Gené’s organ plays a yet unknown role transferring bacteria to tick eggs. This would be a paradigm change, since Gené’s organ has only been known to be involved in the production of a lipidic fluid supporting the survival of tick eggs [66]. Given the intimate involvement of the organ in tick oviposition, its contribution to the core tick microbiome should not be prematurely discarded. Considering the sources of variability reported in this study, the ovary seems to contribute to the microbiota of the tick larvae, presumably in taxa from the female, as well as other sources, such as the spermatophore of the male tick, deposited in the gonopore before the female finishes the blood meal, and this could contribute along with other bacteria. This is a yet unstudied feature, which would ensure an increased variability of larval tick microbiota resulting in the already reported heterozygosity of the tick holobiont [10].

## Conclusions

The ovary of engorged ticks does not seem to be the only source of a core microbiota of the larvae, and a contribution by Gené’s organ or the spermatophore of the male(s) must be considered. The standard method to eliminate the external bacteria in the tick eggs for studies on microbiota has not been adequately standardised to produce a repeatable protocol. Research is necessary to determine (i) whether such “external” bacterial fauna of eggs is only contamination or belongs to the “core” microbiome, protected of exogenous contamination by the lipid surrounding egg masses, and (ii) the effects of bleach on engorged females to avoid undesirable effects on measurements of the microbiota. Suitable statistical analysis should guide conclusions towards a better overview of the changes in bacterial composition. Studies of bacteria known to belong to the tick’s internal milieu should be used as a target for testing the protocols for the removal of external bacteria. Using bleach could be an effective method for tick egg removal of external contaminating microbiota once a suitable protocol has been assayed.

## Supplementary Information

**Additional file 1: Table S1.** Complete table with all the recorded bacteria in the larvae of *Rhipicephalus australis* and the reads in all the groups. Column A corresponds to the microorganism, and the following columns contain the number of reads of each one in the different groups and replicates of treatment. First two rows correspond to external and internal numerations with abbreviations. Each column contains the abbreviation of the treatmentfor females and/or eggsas well as the number of replicates.

## Data Availability

All the data of study are published in Supplementary material.

## Abbreviations

NT: No treatment
UV: Ultraviolet light
B: Bleach
GO: Gené’s organ
ASV: Amplicon sequences variables
PD: Phylogenetic distance
